# Supplementary material for: PatchWarp: Corrections of non-uniform image distortions in two-photon calcium imaging data by patchwork affine transformations
Source: Cell Rep Methods. 2022 Apr 27;2(5):100205. doi: 10.1016/j.crmeth.2022.100205 (PMC9142688; doi:10.1016/j.crmeth.2022.100205)
Supplement: Document S1. Figures S1–S5 and Table S1 [file mmc1.pdf]

Cell Reports Methods, Volume *2*

Supplemental information

PatchWarp: Corrections of non-uniform image distortions in two-photon calcium imaging data by patchwork affine transformations

Ryoma Hattori and Takaki Komiyama

Cell Reports Methods, Volume *2*

Supplemental information

PatchWarp: Corrections of non-uniform image distortions in two-photon calcium imaging data by patchwork affine transformations

Ryoma Hattori and Takaki Komiyama

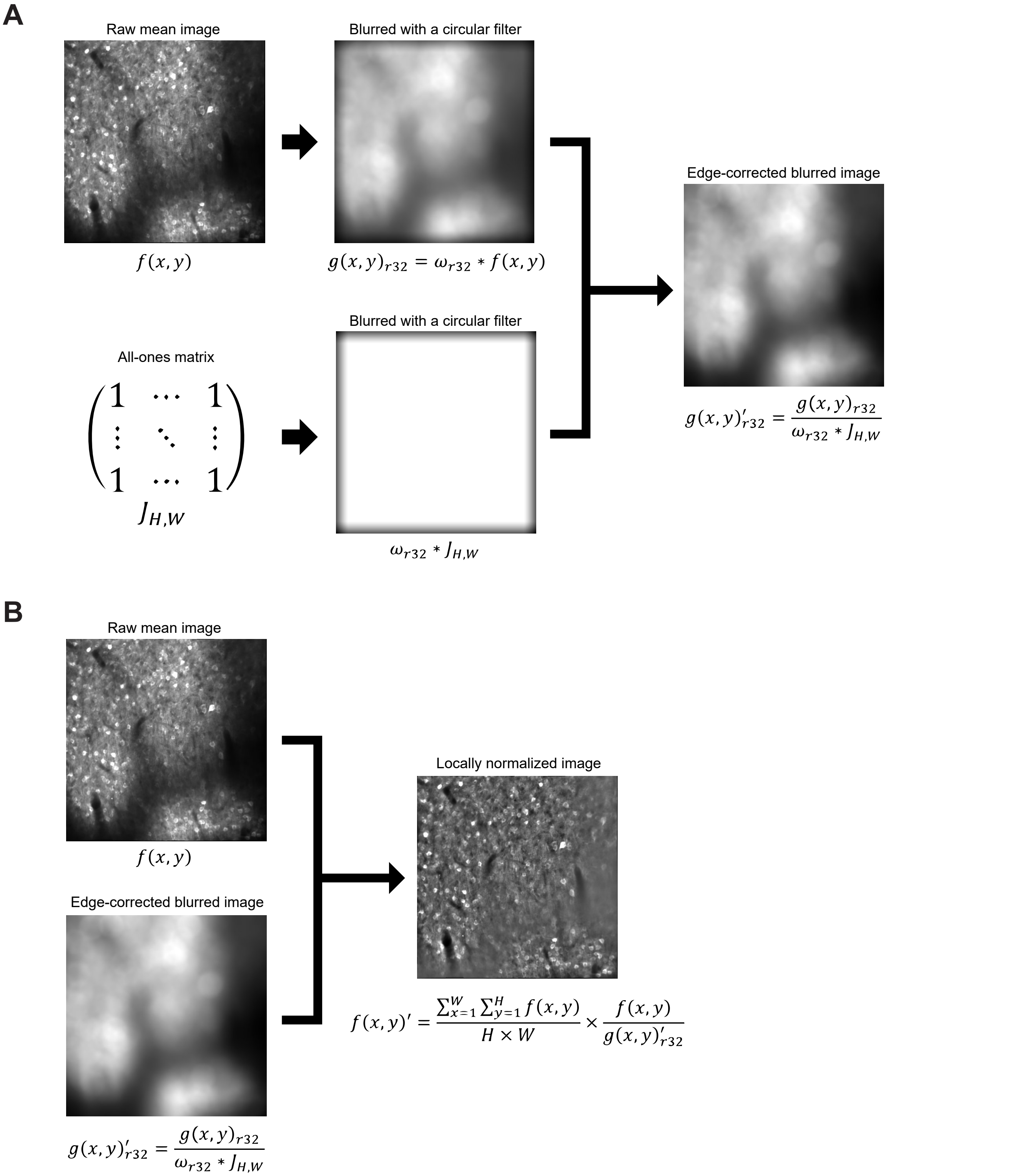

Figure S1. Normalization of mean images by their local intensity, Related to Figure 2.

(**A**) Steps for making a blurred image (local intensity map) which will be used to normalize a mean image. First, the original mean image was convolved by a circular filter with a radius of 32 pixels. The edge pixels are dim due to zero-paddings. To correct the zero-padding effects, we apply the same convolution to all-ones matrix with the same pixel number as the mean image. The resulting convolved all-ones matrix image also exhibits dim intensity near the edges due to zero-paddings. Therefore, division of the convolved mean image by the convolved all-ones matrix results in a blurred image without zero-padding artifacts.

(**B**) Steps to make an image with local intensity normalization. The intensity of each pixel in the blurred image from (A) reflects the local intensity near the pixel. Therefore, simple division of the original mean image by the blurred image normalizes each pixel intensity by its surrounding intensity. To return the intensity value back to the original scale, the division is multiplied by the mean intensity of the original mean image. Note that the two images on the left are duplicates from (A).

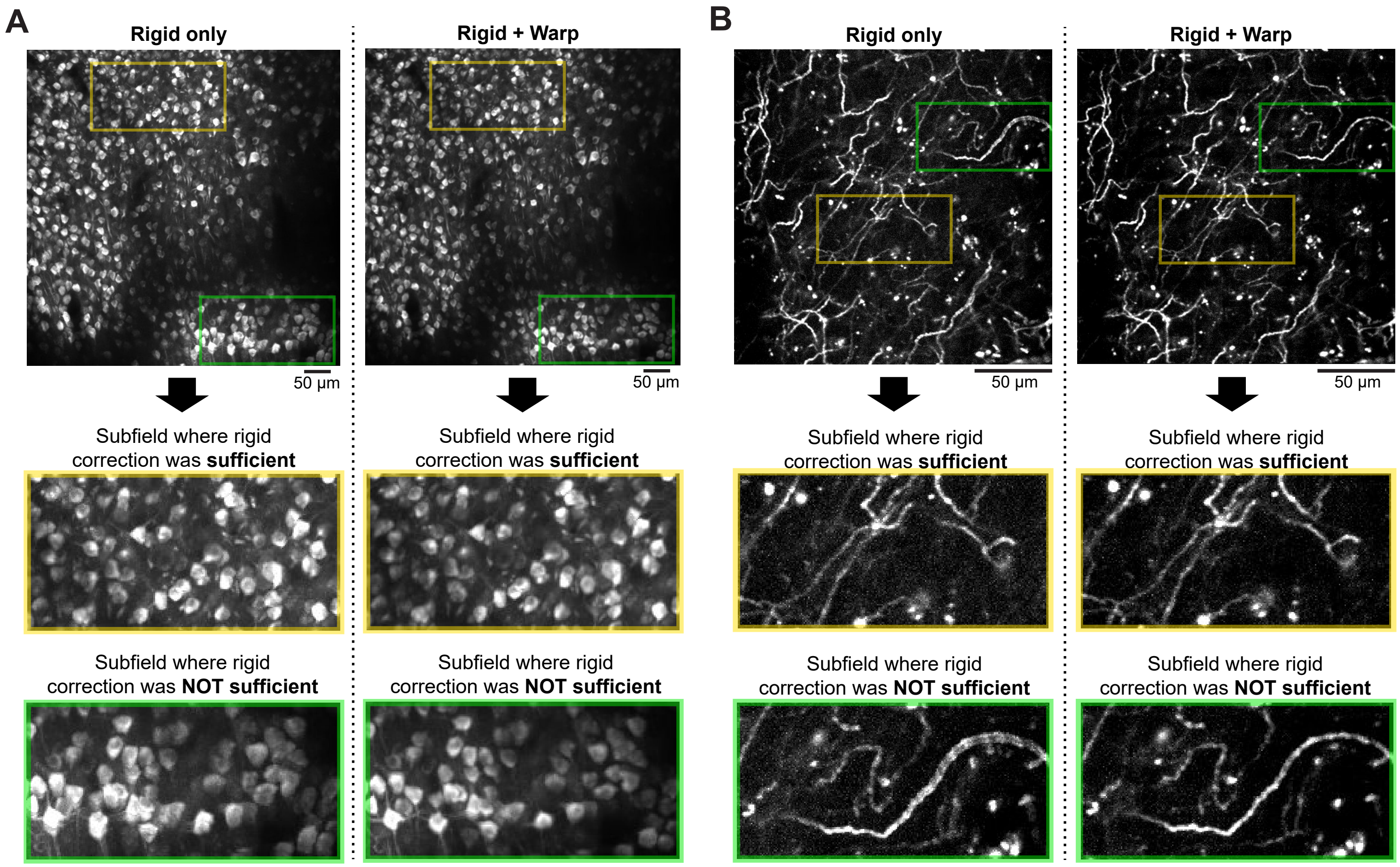

Figure S2. Corrections of non-uniform image distortions on images from Figure 1, Related to Figures 1 and 2.
(**A**) Max-intensity projection image of an example RSC imaging session. The max-intensity projection of all frames after correcting only rigid motion artifacts (Left), and the max-intensity projections of all frames after correcting both rigid motion artifacts and non-uniform distortions by PatchWarp.
(**B**) Max-intensity projection image of an example cholinergic axon imaging session. The max-intensity projection of all frames after correcting only rigid motion artifacts (Left), and the max-intensity projections of all frames after correcting both rigid motion artifacts and non-uniform distortions by PatchWarp.

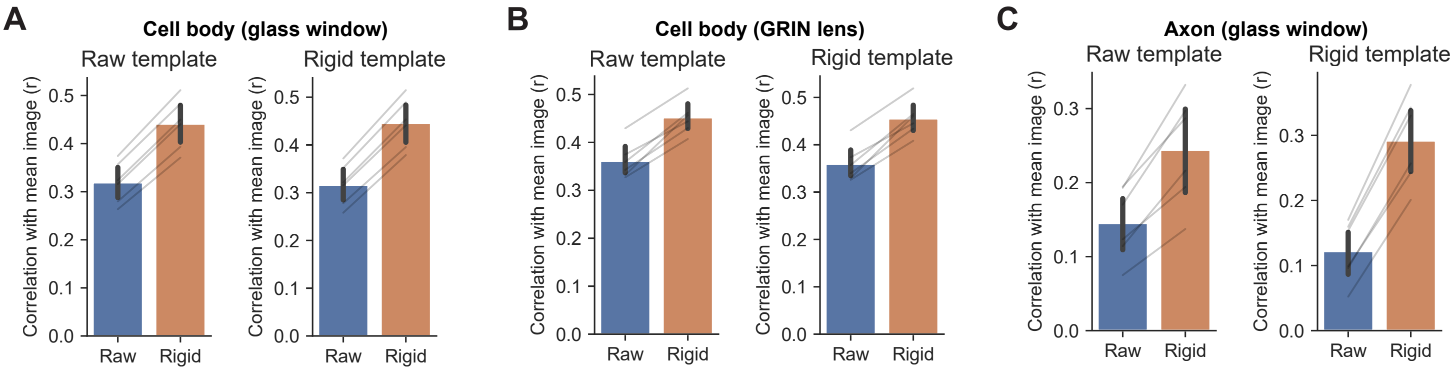

Figure S3. mCM with consistent template images for comparisons between pre- and post-rigid corrections, Related to Figure 3. (**A-C**) Mean correlation between the mean of either raw frames (Raw template) or rigidly corrected frames (Rigid template) and individual frames. mCM consistently increases after rigid motion corrections, regardless of which template image is used to calculate the correlation. Therefore, the difference in self-mCM in Figure 3B is not due to the different template images for Raw and Rigid conditions.

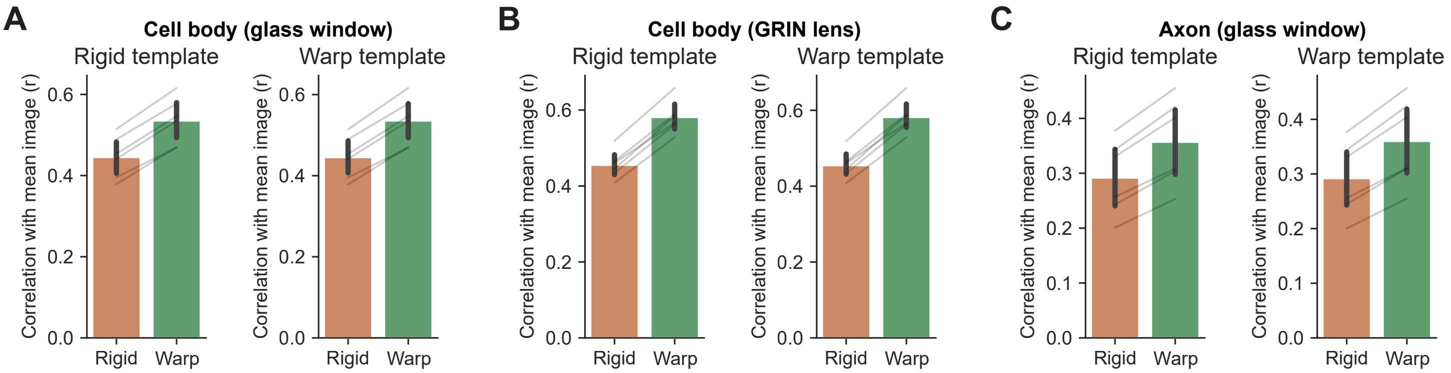

Figure S4. mCM with consistent template images for comparisons between pre- and post-warp corrections, Related to Figure4. (**A-C**) Mean correlation between the mean of either only rigidly corrected frames (Rigid template) or distortion-corrected frames (Warp template) and individual frames. mCM consistently increases after warp corrections, regardless of which template image is used to calculate the correlation. Therefore, the difference in self-mCM in Figure 4B is not due to the different template images for Rigid and Warp conditions.

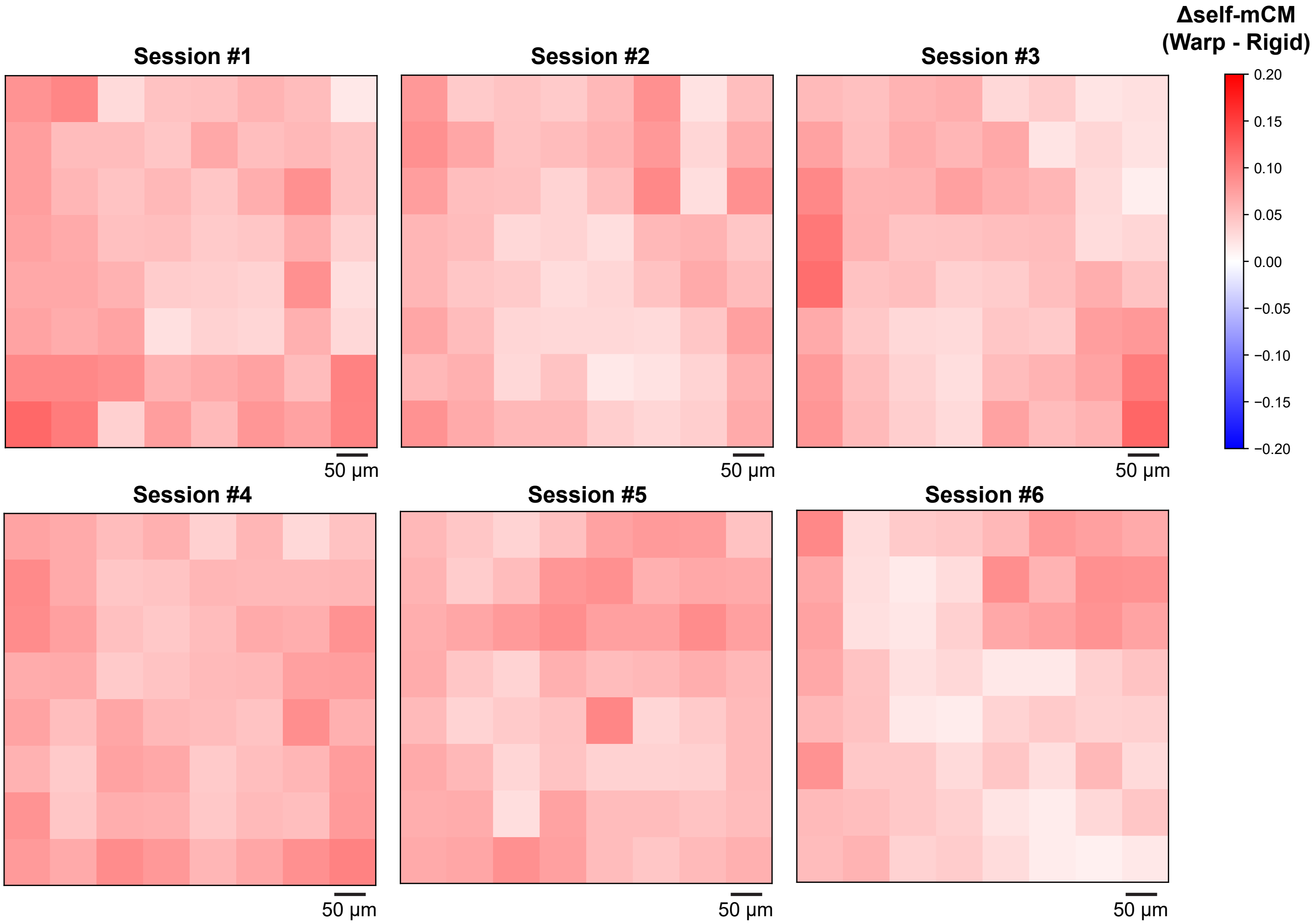

Figure S5. Registration accuracy consistently improves across different patches, Related to Figure4.
Difference in self-mCM for each patch between rigid-only registration and non-rigid registration with warp corrections. Results from 6 imaging sessions (cell body imaging, glass window) are shown. Self-mCM was consistently positive across all patches, indicating that warp correction step consistently improved the registration quality.

Table S1. Comparison of non-rigid registration algorithms for calcium imaging data, Related to Figure 6.

Algorithm	Application in the original paper	Rigid correction	Non-rigid correction
PatchWarp	- Cell body, Glass window, 2-photon<br>- Axon, Glass window, 2-photon<br>- Cell body, GRIN lens, 2-photon	Whole FOV translation (and rotation for across-session registration) with the pyramid representations and their correlations	Piecewise affine transformation with ECC
Suite2p	- Cell body, Glass window, 2-photon<br>- Axon, Glass window, 2-photon	Whole FOV translation with phase correlation	Piecewise rigid translation with phase correlation
CaImAn (NoRMCorre)	- Cell body, Glass window, 2-photon	Whole FOV translation with cross correlation	Piecewise rigid translation with cross correlation
LANMC	- Cell body, GRIN lens, 1-photon	Same as CaImAn	Accelerated piecewise rigid translation of CaImAn with LSTM networks
MIN1PIPE	- Cell body, GRIN lens, 1-photon	Whole FOV translation with Lucas-Kanade method	Diffeomorphic demons
